# The Meeting of Practitioners at Hammersmith

**Published:** 1913-12-27

**Authors:** 


### THE NATIONAL MEDICAL GUILD.

### The Meeting of Practitioners at Hammersmith.

A meeting of medical practitioners in support of the National Medical Guild, which has been registered as a trade union, was held in the Town Hall, Hammersmith, on Friday, December 19, Dr. G. J. Buttar presiding.

The Chairman, in inaugurating the proceedings, said the Council of the Guild had been much impressed recently by two facts. The first of these was that there was trouble looming ahead for the profession, and probably within a very short time; that at all events in the year 1915, if not before, the whole subject of medical benefit under the Insurance Act would once more be under review. And there was the probability, almost amounting to certainty, that in process of time the dependants of insured persons would be included under the Act, and that would mean that thirty-seven millions in these islands would be under the Act. The second point was that, notwithstanding the urgency of this question, there was now very little cohesion in the medical profession, and but little preparation for a future fight. He reminded the audience of the collapse of the schemes suggested by the British Medical Association for the provision of a fund for fighting purposes. These schemes seemed to have been put off for at least seven months, and it was likely that matters of serious import might transpire before then. The invitation to attend this meeting had therefore been sent to confrères over a large area of London, so that the Guild might, if necessary, be criticised, and the attitude of the profession considered. Mr. E. B. Turner had long been a convinced trade unionist; it was he who raised the question at the Liverpool meeting eighteen months ago, and so it seemed fitting to ask him to open this discussion. Following that address, others in the hall would be invited to join in the discussion or to ask questions.

### The Coming Crisis of 1915.

Mr. E. B. Turner said it might be the idea of some members of the profession that a learned profession was not the sort of body to form a trade union; but he had come to the conclusion that under the altered conditions obtaining at the present day it had become an absolute necessity. As the Chairman had well said, they were not at the end of things yet. Some men had been so foolish as to say—and others still more foolish as to write—that the fight for the dignity and the emoluments of the profession was now over, and all that remained was to sit down and make the best—or the worst—of it. That was one of the most unfortunate things which could be said or thought at the present time, because the whole of the relations of the medical profession and those of their patients who belonged to the industrial classes were going to be cast into the melting-pot, and within a very short time, whichever party happened to be in power. Consideration was even now being given to the question of the provision of medical attendance to uninsured people and the dependants of those at present insured. Therefore the profession should take warning from what had passed, and be better prepared for the next crisis than it had been for the last. There was not very much time, because in 1915 the extra 2s. 6d. offered by the Chancellor would come to an end. It was experimental; it was money given in addition to the actuarial calculations on which the Insurance Act was founded. Therefore, during 1915 there would be Bills and Amending Acts on the same subject. There was also another factor which was going to bear very largely on what might be done at the present time—namely, the solvency or otherwise of the approved societies. Those societies were finding under the Insurance Act that there was what they regarded as an abnormal—but what the profession regarded as a normal, having regard to the kind of lives many of them were—rate of sickness, so that their funds for sick pay were seriously decreasing, even though the country had been singularly free from epidemics or accidental cripplings. And in the larger societies it was only by strictest economy, watching every farthing of expenditure, that they were keeping within the range of actuarial safety. If they were likely to come to grief there would be raised a great outcry, and it would be said that the doctors were being paid too much, and that they were not doing enough work for their money. The question of the thirty-seven millions of people who would have to be provided for directly concerned every practitioner whose practice touched the industrial classes. It was therefore most important that the profession should stand up for itself and unite against the advent of these troubles; and it was essential that there should be money available for the purpose. One of the chief causes of having come to grief in the last struggle was that the funds available were not sufficient. There was in January a fund guaranteed by members of the profession, which was vested in the trustees appointed by the British Medical Association. That sum was never more than £137,000, and it was not enough to do what was wanted. Even if men had stood out and it had come to a fight, he doubted if the fund could have been used effectually, even in the way in which the profession tried to safeguard it. Judges had an inconvenient way of brushing aside subterfuges when people appeared before them in a court of law, and, if they had fought, every artifice and means would have been arrayed against them to defeat their object. The judges would have said this was a fund which was not the British Medical Association's, but was organised by them, which was vested in trustees, which trustees were the Council of that Association for the time being, or any committee to whom the Association might choose to delegate its powers. And if the fight had gone against the Association the reserve funds of the Association might have been liable for damages or costs.

### Need for a Safe Fighting Fund.

Wherefore it was clear that if there was to be a fighting fund that fund must be safe, and the only way to make it safe was to make it the fund of a trade union. In this country at present there were only two institutions which were above the law: one was the King, individually, and the other the trade unions, collectively and individually. One had seen instances during the last few weeks of the straining of the law to help trade unions. That was the first strong reason why there should be a trade union in the medical profession to meet whatever might occur in connection with it during the next three years. The ideal, as he had said before, would be for the British Medical Association itself to be a trade union, with its 24,000 members. If that were to come about it could carry on its scientific work on one side and its medico-legal defensive work on the other. But when that problem was approached it was found that the legal difficulties in the way would be almost insuperable. But in advocating a trade union he did not want to be pulling against the British Medical Association; he wanted the trade union to work in harmony with the parent body—to engage on parallel lines. He knew the question of the special fund not having been carried at the last representative meeting was because four representatives considered the fund was not safe vested in trustees appointed by the Association. The matter was now postponed, anyhow, until next July. Even if at that date it should be agreed to there would be no time and very little money for buckling on their armour again. Even now, with its limited numbers, probably the fund of the Guild would be safer than the guarantee fund of the British Medical Association had been during the last two years. In a fight it was sometimes necessary to hit sharp and hard, as well as to defend; the profession could not afford to lower its shield for a month or two while the enemy took pot shots at its members. It was necessary to leave power in the hands of the executive to enable them to take at once such steps as might seem good to them, though of course there must be a ballot of members on such a serious matter as the decision whether there should be a strike. It had been suggested that trade-union funds were not really as safe as they were supposed to be; that Mr. Justice Darling suggested that if one could not sue the union one could sue the individuals of it. That might or might not be so, but he wanted it to be clearly understood that, whatever might happen in regard to individuals, the funds of the union were safe and above the law. Individuals might not particularly mind being killed, and officials had been known to take office well realising that they were to be made cockshies of; but the funds of the union as such would be safe. A further point was that if the profession wanted to get anything done in Parliament it ought to have a voice in Parliament which should speak for the profession and be independent of party.

### The Position of Medical M.P.s.

The members of the profession now in the House of Commons subordinated their profession absolutely to their party; they were ready to go as far for their profession as they could, provided it did not clash with the interests of party. But as they were elected as party men, not as representing the profession, he could find no fault with them. A trade union could have such representatives, for it was empowered to raise the necessary election and other expenses, and even to pay a salary. That might be possible for the Marylebone Division. Such men should be recognised in the House as authorities on all matters appertaining to the medical profession, as well as questions of public health and hygiene. The question affected those who were working under the Insurance Act, because they would be put in the forefront of the battle when the reduction of the expenses and the bankruptcy of the friendly societies came forward. It appealed to the whole profession, because there was a very strong feeling among certain sections of politicians that the best way of looking after the health of the United Kingdom would be a complete and thorough State Medical Service, starting from almost the dispensary and ending with the senior consultant. He personally would look upon such a thing as most injurious, both to the profession and to the health of the public; and if it desired, the profession, when thoroughly organised, could put an effective stop on any such project. In advocating a trade union, he wished it to be one open to every member of the profession. He wanted the profession to be one and indivisible, which would work in perfect amity, and alongside and on parallel lines with, the British Medical Association. (Much applause.)

The Chairman invited a general discussion.

### The General Discussion.

Dr. Boclet was sure the meeting had listened with great interest to Mr. Turner's address. He knew the great difficulties which Mr. Turner had. There was first the great apathy shown by the profession, an apathy which was appalling. He noticed it at a divisional meeting of the British Medical Association at Westminster. The Association had party government, legislation, wrangling between lawyers, and all the professional misfortunes and sorrows, and in this abyss they looked for a man to lead them and save them from extinction. The right man had evidently been found in Mr. Turner. The profession had been humiliated in the eyes of the public. A business man said to him in the City that commercial men looked to the medical profession at the time the National Insurance Act was being projected to do something, for that profession had the reins in its hands, but they did not stand together. He asked Mr. Lloyd George's doctor why he did not stand with them, and the reply was that it was a delicate matter for him, as he attended Mr. Lloyd George and his family. (Laughter.) He did not propose to censure the British Medical Association, because he believed their failure to do what was wanted was due to lack of finance. He appealed to the common sense of the profession, so that they might stand united. Whether the organisation were called a trade union or not, it was necessary for the profession to be prepared. At present it was in a parallel condition to the routed Turks.

Dr. Tompkins said that although he had been asked to lead the opposition against Mr. Turner's position, he felt himself unable to do so, largely because his views almost entirely accorded with those of that gentleman. But the British Medical Association had been blamed for this failure. One or two members of the Council deserted them in time of need. Who were the Association? They were the units of the profession, and the absolute apathy of the members at the time of the crisis was the cause of nothing more being done. (Applause.) They did not even attempt to get together a decent fund. The excuses which one heard at the time were enough to make one feel disgusted with one's profession. All that was asked was that members would give two or three pounds. Most of them wasted more than that in cigarettes in a year, yet seemed unwilling to deprive themselves of any indulgence to safeguard their profession. If the units of a body were content to leave all the work to the Executive there would be failure. Why was it that trade unions among the workers of the world had gained such an ascendancy? It was because of the personal interest of the individual units. Possibly they did not go on to platforms and speak, but they talked to one another at the gates as they left their work, and it was clear that each member had an interest—a personal interest—in what was going forward. The British Medical Association was the oldest and the largest combination of medical men, and it was the fault of the members if that Association, as such, had not in the past stood the profession in good stead. Over a year ago he advocated that instead of small Associations being formed, those men who were going to form the Executives of the Associations should band themselves together to capture the British Medical Association, and bring it into line with what was felt to be the opinion and wish of the profession at large. That seemed to have been a failure, but he could not help thinking that the British Medical Association was still the body to which they should turn, and he was therefore glad to hear Mr. Turner say he hoped they would work in unison with the Association. He hoped they would do more than that, and that when the Medical Guild comprised a sufficient number of members to make its voice powerful they would endeavour to get the British Medical Association to work in harmony with it, so that the strictly trade-union work of the profession could be handed over to the Guild through the committees of the British Medical Association, so that thereby the British Medical Association might be holding the strings of all the other Societies, instead of opposition on the part of the newly formed bodies to the parent Association.

### The Marquis of Zetland *v.* The B.M.A.

Dr. Percy C. Raiment (General Secretary) reminded the meeting that the Association started last January, and he had now the honour of being one of the Executive officers. A resolution which would be submitted later expressed the hope that the British Medical Association would recognise the Guild as an auxiliary body, so that it would be clear that the Guild was not antagonistic to the British Medical Association. The Guild arose about the old London Medical Committee, which did excellent—or otherwise, according to the point of view—work when the Insurance Act came in. The Guild was launched with very high hopes and a very small purse in April this year. It was registered in July, and the Certificate of Registration was evidence in any Court that the body was a trade union. And if for any purpose it was desired to plead Section 4 of the Trades Disputes Act, it would not be necessary to argue whether or not the body was a trade union; the certificate made it clear. He mentioned that because there was talk in some quarters of an unregistered trade union. An unregistered trade union would labour under the enormous disability of having to prove that it was a *bona-fide* trade union. Trade unions possessed certain definite legal immunities and privileges. The funds of the union, by the Trades Disputes Act of 1906, were protected from actions for libel, slander, actions in restraint of trade, or actions concerning conspiracy or tortious acts. Tortious acts had been defined by a lawyer as anything from pickpocketing to manslaughter, so there was a very wide range over which the funds of the union were absolutely immune. Of course, it would be a sorry day for the profession if it were felt there would be privilege attached to libelling or slandering a fellow-practitioner. The question of restraint of trade was the bedrock of trade unionism. The British Medical Association could not act in restraint of trade, as witness the case of the Marquis of Zetland versus The British Medical Association. The Marquis of Zetland was seeking an injunction to restrain the British Medical Association from publishing certain matters and passing certain resolutions, because they were interfering with the free employment of labour in the market. Of the merits of the quarrel he knew nothing, and he did not propose to discuss them; but the matter had been adjourned to allow the parties to come to terms. If the decision should go one particular way, then the excellent work which had been carried on by the *British Medical Journal* for many years, in issuing warning notices concerning barred appointments, would cease; the Journal would not be in a position to warn the profession that certain appointments were barred. The action had come at a most opportune moment to prove what they of the Guild had been preaching up and down the country during the last six months.

### What Strength has the Guild?

With regard to the strength of the Guild, the number of its members seemed to be limited by the time which he and his colleagues could devote to the work. In September some of them went to the Salford Division of Manchester, and the result was beyond all their expectations. The profession welcomed them enthusiastically, and now he believed there were not 5 per cent. of the practitioners there who were not members of the Guild. Next month he would be calling a meeting of over 2,000 in Manchester, at the express invitation of the Joint Committee of the British Medical Association in Manchester, and Dr. Arthur Helm would be in the chair. He believed that by the end of January Manchester would be theirs. Branches had been established in Sheffield, Leeds, Manchester, Oldham, Rochdale, Chester, Plymouth, South Wales, Forest of Dean, on the South Coast, and in Essex, and they were all increasing. With regard to numbers, it was necessary to be careful, not because one did not desire to speak the truth, but because there were two ways of reckoning numbers. He would give an instance. In the Isle of Wight he thought there were six members. At the representative meeting he found the secretary had been so busy that instead of six there were forty. He had now deposited in the office membership forms for 1,200, and they included some of the most important members of the British Medical Association. The whole essence was that of the funds, and if the funds were to be protected it was necessary to have a trade union. That was not his own unsupported opinion. In the past an old guarantee fund was raised by voluntary effort, and it was found that the same people gave each time. The method of the Guild was to raise the necessary funds by levies. Those were really semi-compulsory. When a practitioner was a member he must pay any levy required, unless he could show good cause why he should not. If he could not show such good cause, he must leave the organisation. Powers had been taken to levy up to £20 in any one year. That had startled many men, but there was not a likelihood that anything like that amount would be levied. In the piping times of peace there would accrue quite a good sum. Three peaceful years would mean, on a reasonable computation, £200,000. He put it to the meeting whether they were willing to go on another six months waiting for something—they knew not what—waiting for the British Medical Association to evolve some scheme, which in the end would possibly not result in protection such as a trade union would bring?

### Three Possible Courses.

Could the profession afford to wait, and throw away the first seven months of 1914? For eighteen months they had been trying to get the British Medical Association to see the importance of the matter, but they would not touch it, and that was still their attitude. There were three possible courses: (1) Turning the British Medical Association into a trade union; Mr. Turner and the solicitors had told them that was almost a legal impossibility. (2) To have a trade union, the Council of which was the Council of the British Medical Association for the time being.

But no Registrar of Trade Unions would pass a set of rules which included such a rule as this, for the essence of trade unionism was democratic government. Moreover, it was compulsory to have a rule concerning alterations of rules, and it would be open to a trade union to call an extraordinary general meeting and alter its rules and throw the Council of the British Medical Association overboard. (3) To found a fresh trade union outside the Association, but certainly in harmony with it, so that the funds could be protected for the carrying out of the medico-political work, leaving the British Medical Association to do the scientific work. That third course had been followed.

Dr. Burnhill emphasised that the dominant note of the promoters of the Guild was that they were acting in conjunction with, and not in opposition to, the British Medical Association. At an earlier meeting at Kensington Town Hall, he was the first to make mention of the term trade union. He urged that the new body should give the profession a distinct lead. If the British Medical Association was beyond redemption, practitioners might as well sever their connection with it and devote their energies to a proper trade-union organisation. He objected to the remark about the apathy of the profession, the fact being that they were badly led at the time of the Insurance Act. If it were to be understood that everything must be done in conjunction with the British Medical Association he could not see the advantage of joining it. (Applause.) In 1915 a similar crisis would probably have to be encountered to that already passed through. The profession only represented 30,000 votes, and the only hope would be to stand unitedly.

### Text of the Resolution.

Dr. Townsend proposed: "That this meeting of practitioners, having heard Mr. E. B. Turner, and being convinced of the necessity of a trade union for the profession, pledges itself to support the National Medical Guild, and to use every legitimate means to compel the British Medical Association to recognise the Guild as an auxiliary body." The Guild, he said, could do everything for the general practitioner, and do it quickly. He gave an instance in his own practice of the unsatisfactory delays when a question touching professional ethics arose. It was the fault of an antiquated method. The salvation of the profession lay in its own hands. No hope could be expected from the political parties. It was realised that the British Medical Association as at present constituted could not act speedily enough. It took so long to turn in its lair that the quarry had escaped before it was well awake. They wanted the British Medical Association to say to the Guild, "You look after the finances and the trade-union aspect of the profession, and we will do the scieintific work."

Dr. Raiment seconded the resolution.

Dr. Burnhill said he took it that the resolution was really asking for the British Medical Association's paternal blessing.

The resolution was carried with three dissentients.

The thanks of the meeting were tendered to Mr. Turner for his address, and Mr. Turner, in thanking the meeting for the compliment, invited his hearers to cast their eyes across the Channel, where the Germans were fighting a similar battle and would probably get what they wanted. That was simply because they possessed a proper trade union, embracing all the men who were affected. That constituted a good example for the profession in England to follow.

